# Empagliflozin Targets NF-κB Signaling Through PTGS2 and TLR4 in Polycystic Ovary Syndrome: A Drug Repurposing Study and Molecular Simulation

**DOI:** 10.3390/jox16050169

**Published:** 2026-09-07

**Authors:** Ikhwandi Chandra Nugraha, Ami Febriza, Asdar Tajuddin, Suryani As’ad

**Affiliations:** 1 Department of Medicine, Muhammadiyah University of Makassar, Makassar 90221, Indonesia; ikhwandichn@med.unismuh.ac.id; 2 Department of Physiology, Muhammadiyah University of Makassar, Makassar 90221, Indonesia; amifebriza@med.unismuh.ac.id; 3 Department of Surgery, Muhammadiyah University of Makassar, Makassar 90221, Indonesia; asdarhikari@med.unismuh.ac.id; 4 Department of Nutrition, Hasanuddin University, Makassar 90245, Indonesia

**Keywords:** empagliflozin, polycystic ovary syndrome, network pharmacology, molecular simulation, drug repurposing

## Abstract

Polycystic ovary syndrome (PCOS) is a multifactorial endocrine disorder characterized by chronic inflammation, insulin resistance, and reproductive dysfunction. Although empagliflozin, a sodium-glucose cotransporter-2 inhibitor, has demonstrated anti-inflammatory and metabolic benefits, its molecular mechanisms in PCOS remain poorly understood. This study investigated the anti-inflammatory mechanisms of empagliflozin in PCOS using network pharmacology, molecular docking, and molecular dynamics simulations. Potential drug targets were identified using SwissTargetPrediction and SuperPred, while PCOS- and inflammation-related genes were obtained from GeneCards. Overlapping targets were subjected to Gene Ontology, Kyoto Encyclopedia of Genes and Genomes, protein–protein interaction network, and hub gene analyses. Molecular docking and 50 ns molecular dynamics simulations were performed to evaluate binding affinity and complex stability. Key inflammatory targets identified included TNF, IL6, IL1B, TLR4, STAT3, and PTGS2, with significant enrichment in cytokine-mediated signaling, TNF signaling, and NF-κB pathways. Empagliflozin showed strong binding affinities for PTGS2 (−9.0 kcal/mol) and TLR4 (−8.8 kcal/mol), while molecular dynamics simulations demonstrated stable protein–ligand complexes throughout the simulation. These findings suggest that empagliflozin may alleviate PCOS-associated inflammation by modulating the TLR4/NF-κB/PTGS2 signaling axis, supporting its potential as a repurposed therapeutic agent for PCOS and providing a foundation for future experimental validation.

## 1. Introduction

Polycystic ovary syndrome (PCOS) is a complex endocrine and metabolic disorder characterized by hyperandrogenism, chronic ovulatory dysfunction, and polycystic ovarian morphology affecting women of reproductive age [1]. PCOS not only affects reproductive function but is also associated with metabolic disturbances, such as insulin resistance, obesity, and dyslipidemia, as well as an increased risk of type 2 diabetes mellitus and cardiovascular disease [2]. Pathophysiologically, PCOS involves complex interactions between hormonal imbalance, chronic inflammation, and insulin resistance, which lead to ovarian dysfunction and systemic metabolic disturbances [3].

The Global Burden of Disease (GBD) 2021 analysis shows that the global burden of PCOS has increased significantly over the past three decades. Globally, the number of women living with PCOS increased from 36.7 million cases in 1990 to 69.5 million cases in 2021. During the same period, the incidence of PCOS also increased from 1.5 million to 2.3 million cases, accompanied by an increase in years lived with disability from 323,799 to 607,757. These findings emphasize that PCOS is not only a reproductive issue but also a global public health burden [4].

The reported prevalence of PCOS varies substantially across populations and diagnostic criteria. In Asian populations, prevalence estimates have differed across studies, reflecting differences in diagnostic criteria, study populations, and geographic settings [5]. Current PCOS therapy generally focuses on symptom management through lifestyle modification and the use of drugs such as metformin, combined oral contraceptives, and antiandrogen agents to improve insulin resistance and hormonal imbalance [6].

However, the effectiveness of these conventional therapies remains limited, as some patients show suboptimal responses, particularly in cases associated with obesity and severe insulin resistance [7]. One promising approach is drug repurposing, which refers to the reuse of existing drugs developed for other diseases as new therapeutic options for different conditions [8]. In this context, sodium-glucose cotransporter-2 (SGLT2) inhibitors, originally developed for the treatment of type 2 diabetes mellitus, have gained attention as potential therapeutic candidates for PCOS [9,10], These agents work by inhibiting glucose and sodium reabsorption in the renal proximal tubules, thereby increasing urinary glucose excretion and improving insulin sensitivity [10].

Several recent studies have shown that SGLT2 inhibitors can reduce body weight, improve insulin resistance, and enhance metabolic profiles in women with PCOS [11]. Empagliflozin is a novel hypoglycemic agent belonging to the SGLT2 inhibitor class [12]. Empagliflozin has demonstrated efficacy in improving glycemic control, reducing body weight, and enhancing metabolic parameters in patients with type 2 diabetes. Emerging evidence also suggests that empagliflozin exhibits anti-inflammatory and antioxidant properties, which may be beneficial in mitigating the chronic low-grade inflammation commonly associated with PCOS [13]. In experimental PCOS models, empagliflozin has been reported to ameliorate metabolic disturbances and attenuate inflammatory and oxidative stress responses, which are closely associated with ovarian dysfunction, thereby suggesting its potential therapeutic role in the management of PCOS [14].

Despite these promising findings, studies specifically investigating the mechanistic role of empagliflozin in PCOS remain limited, warranting further exploration of its therapeutic potential. Therefore, this study aims to investigate the anti-inflammatory effects and underlying mechanisms of empagliflozin in PCOS. A combination of computational approaches, including network pharmacology and molecular simulation, will be employed to systematically elucidate the molecular targets and signaling pathways involved. In summary, this study seeks to bridge the existing research gap by providing a comprehensive understanding of the mechanistic basis of empagliflozin in PCOS. The findings are expected to offer novel insights into the potential repurposing of empagliflozin as a therapeutic strategy for women with PCOS, thereby contributing to improved disease management.

## 2. Materials and Methods

### 2.1. Network Pharmacology

The pharmacological activity spectrum and potential adverse effects of empagliflozin were initially predicted using the PASS online tool (Way2Drug, version 2.0; http://www.way2drug.com/passonline, accessed on 11 March 2026), which estimates the probability of biological activities based on the chemical structure of the compound [15]. Potential targets associated with PCOS were retrieved from the GeneCards database (version 6.1; https://www.genecards.org, accessed on 11 March 2026) using the keyword “polycystic ovary syndrome.” Inflammation-related targets were identified using the keywords “inflammation” and “anti-inflammatory,” and only targets with a relevance score ≥10 were included to ensure reliability [16]. The PCOS-related and inflammation-related gene sets were initially maintained as separate datasets. After standardization of gene symbols and removal of duplicate entries within each dataset, a three-way intersection was performed between the empagliflozin target set, PCOS-related genes, and inflammation-related genes. Genes present in all three datasets were retained as candidate targets for subsequent enrichment and network analyses.

The putative targets of empagliflozin were predicted using SwissTargetPrediction (2019 version; http://www.swisstargetprediction.ch/, accessed on 11 March 2026) and SuperPred (version 3.0; https://prediction.charite.de/index.php, accessed on 11 March 2026). The overlapping targets between the drug and disease datasets were identified using Venny (version 2.1) and these intersection targets were considered potential therapeutic targets [17]. Subsequently, the intersecting targets were subjected to Gene Ontology (GO) enrichment analysis and Kyoto Encyclopedia of Genes and Genomes (KEGG) pathway analysis using ShinyGO (version 0.85) [18]. To investigate the interactions among these targets, a protein–protein interaction (PPI) network was constructed using the STRING database (version 12.0; http://string-db.org/, accessed on 11 March 2026). Finally, key hub genes were identified using the CytoHubba plugin (version 0.1) in Cytoscape (version 3.10.4) based on topological analysis [19]. Hub genes were ranked using the maximal clique centrality (MCC) algorithm implemented in CytoHubba, and the top-ranked genes were considered hub targets.

### 2.2. Molecular Docking

Molecular docking simulations were conducted to evaluate the binding interactions between empagliflozin and the identified hub target proteins. The three-dimensional (3D) structures of the target proteins were obtained from the RCSB Protein Data Bank (PDB) (http://www.rcsb.org/, accessed on 11 March 2026) [20]. The 3D structure of empagliflozin was retrieved from the PubChem database (https://pubchem.ncbi.nlm.nih.gov, accessed on 11 March 2026) and prepared for docking analysis [21].

Prior to docking, protein structures were processed by removing water molecules and co-crystallized ligands, followed by the addition of polar hydrogens. The ligand structure was energy-minimized to obtain a stable conformation. Molecular docking simulations were performed using the PyRx virtual screening tool (version 0.8; https://pyrx.sourceforge.io/, accessed on 11 March 2026), which integrates AutoDock Vina (version 1.1.2) for predicting ligand–protein binding affinity. Blind molecular docking was performed by maximizing the docking search space to allow exploration of potential binding regions across the target protein without imposing a predefined binding site. The resulting docking poses were ranked according to their predicted binding affinity (kcal/mol), and the top-ranked pose with the most favorable docking score was selected for subsequent protein–ligand interaction analysis using BIOVIA Discovery Studio Visualizer.

### 2.3. Interaction Analysis Using BIOVIA Discovery Studio

The molecular interactions between empagliflozin and the selected target proteins were further analyzed and visualized using BIOVIA Discovery Studio Visualizer (version 2025; Dassault Systèmes, San Diego, CA, USA; https://3dswym.3dexperience.3ds.com/wiki/biovia-drug-discovery-development/free-download-biovia-discovery-studio-visualizer_guJYb_aPRESHev6_XjAhCQ, accessed on 11 March 2026) [22]. The best docking conformations obtained from PyRx (AutoDock Vina) were selected based on the lowest binding energy and appropriate binding orientation within the active site [23].

The docked complexes were subsequently imported into Discovery Studio for interaction analysis. Key ligand–protein interactions, including hydrogen bonds, hydrophobic interactions (alkyl and π-alkyl), van der Waals forces, and π–π stacking interactions, were identified [24]. The amino acid residues involved in binding within the active site were documented for each complex. Both two-dimensional (2D) and three-dimensional (3D) interaction diagrams were generated to visualize the binding modes and to better understand the stability of the ligand–protein complexes. These analyses were used to support the docking results and to elucidate the potential molecular mechanisms underlying the anti-inflammatory activity of empagliflozin.

### 2.4. Molecular Dynamics

The dynamic stability of the TLR4–empagliflozin and PTGS2–empagliflozin complexes was evaluated using molecular dynamics (MD) simulations performed with YASARA Dynamics version 4.3.13 (YASARA Biosciences GmbH, Vienna, Austria). Based on the molecular docking results, both complexes were selected for simulation due to their favorable binding affinities and biological relevance. Hydrogen atoms were added, and each system was solvated in an explicit TIP3P water box and neutralized with 0.9% NaCl. Following energy minimization, MD simulations were conducted for 50 ns using the AMBER14 force field under periodic boundary conditions at 298 K and 1 atm. Long-range electrostatic interactions were calculated using the particle mesh Ewald method. The structural stability of each complex was evaluated by analyzing the root mean square deviation (RMSD), root mean square fluctuation (RMSF), radius of gyration (Rg), and solvent-accessible surface area (SASA) throughout the simulation [25,26]. The structural behavior of the protein–empagliflozin complexes was evaluated from the molecular dynamics trajectories using RMSD, RMSF, radius of gyration (Rg), and SASA analyses. RMSD was calculated to evaluate the overall structural deviation of each protein–ligand complex relative to its reference structure throughout the simulation. RMSF analysis was performed on a residue-by-residue basis to characterize local fluctuations and identify regions with greater structural flexibility. The radius of gyration (Rg) was analyzed to assess changes in the overall compactness of the protein structure during the simulation. SASA was calculated to evaluate changes in the solvent-exposed surface area of the protein–ligand complexes. The resulting trajectories were analyzed throughout the 50 ns simulation period, and the corresponding profiles were used to evaluate the overall conformational behavior of the complexes [25].

## 3. Results and Discussion

### 3.1. PASS Prediction Analysis

The PASS prediction results (Table 1) demonstrated that empagliflozin possesses a high probability of antidiabetic activity (Pa = 0.850), which is consistent with its established pharmacological role as a SGLT2 inhibitor. In addition to its primary activity, empagliflozin also exhibited moderate probabilities of anti-inflammatory and antioxidant activities, suggesting that it may exert pleiotropic effects beyond glucose regulation. These findings are particularly relevant in the context of PCOS, which is increasingly recognized as a chronic inflammatory and metabolic disorder. Oxidative stress and inflammation are known to contribute to ovarian dysfunction, insulin resistance, and hyperandrogenism in PCOS. Recent studies have reported that SGLT2 inhibitors can reduce oxidative stress markers and inflammatory cytokines, thereby improving metabolic balance.

### 3.2. Network Pharmacology Analysis

#### 3.2.1. Identification of Intersection Targets

**Figure 1:**
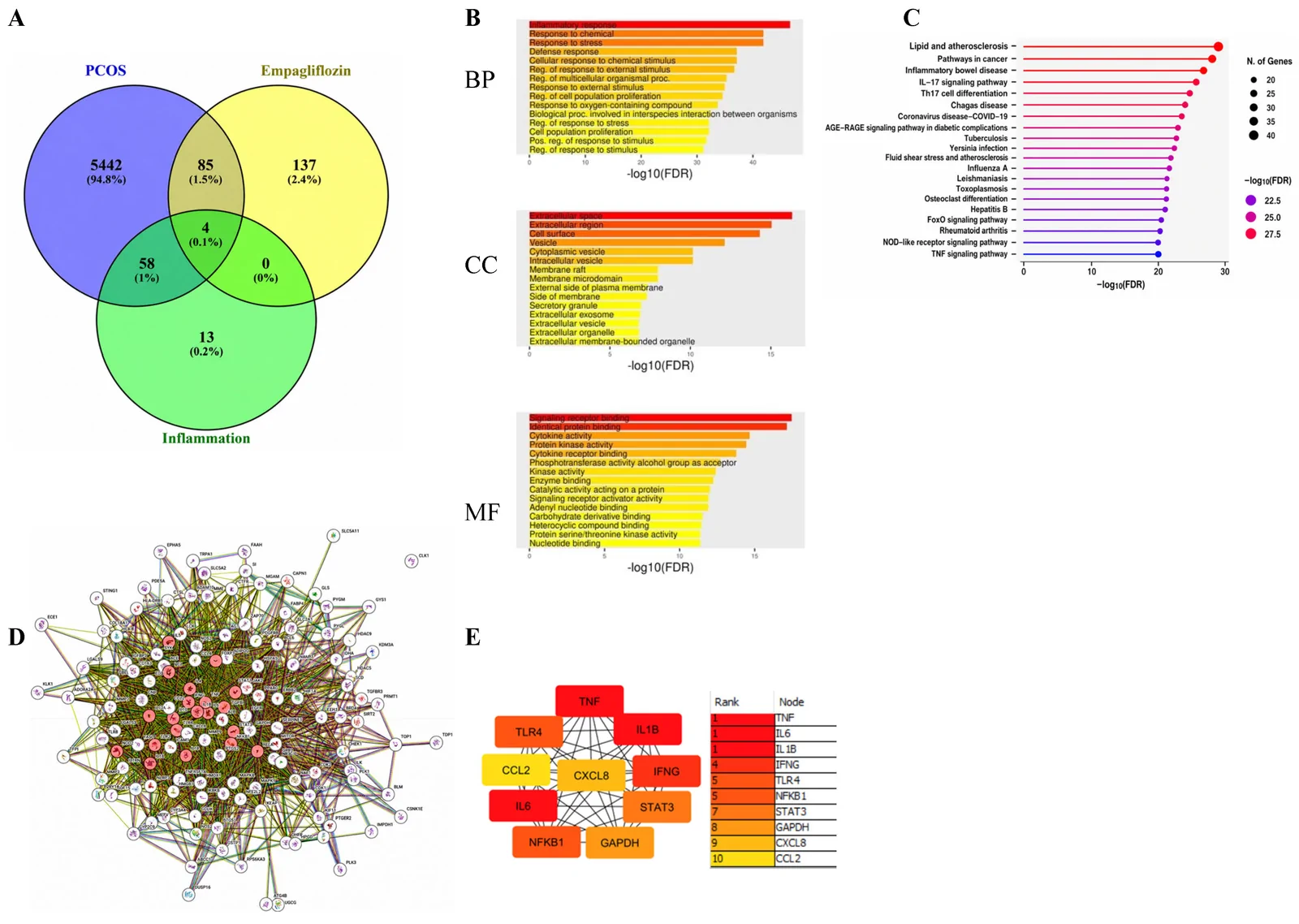
Identification of core targets of empagliflozin in PCOS. (**A**) Venn diagram of empagliflozin, PCOS, and inflammation targets; (**B**) GO enrichment analysis; (**C**) KEGG pathway analysis; (**D**) PPI network of intersecting targets; (**E**) hub genes identified by CytoHubba. In panel (**B**), the color gradient represents −log10(FDR), with red indicating higher values and yellow indicating lower values. In panel (**E**), node colors indicate hub-gene ranking based on the maximal clique centrality (MCC) algorithm, with red representing the highest-ranked genes (ranks 1–4), orange representing intermediate-ranked genes (ranks 5–7), and yellow representing lower-ranked genes (ranks 8–10).

The Venn diagram analysis (Figure 1A) revealed overlapping targets among empagliflozin, PCOS, and inflammation datasets, indicating potential therapeutic targets through which empagliflozin may exert its effects. This overlap suggests a multitarget pharmacological profile, which is particularly important for complex diseases such as PCOS that involve interconnected metabolic and inflammatory pathways. The ability of a compound to act on multiple targets is considered advantageous in systems pharmacology, as it allows simultaneous modulation of several dysregulated biological processes.

#### 3.2.2. GO and KEGG Enrichment Analysis

The GO enrichment analysis (Figure 1B) indicated that the intersecting targets are primarily involved in inflammatory and immune-related processes, particularly cytokine-mediated signaling pathways. This finding highlights the central role of inflammation in PCOS pathogenesis. Furthermore, KEGG pathway analysis (Figure 1C) revealed significant enrichment in pathways such as TNF signaling, NF-κB signaling, and cytokine–cytokine receptor interaction. These pathways are known to contribute to insulin resistance and chronic inflammation. Activation of the NF-κB pathway promotes the expression of pro-inflammatory cytokines, thereby exacerbating metabolic disturbances in patients with PCOS.

#### 3.2.3. PPI Network and Hub Gene Identification

The protein–protein interaction (PPI) network (Figure 1D) demonstrated a highly interconnected network of target proteins, indicating strong functional relationships among them. Subsequent CytoHubba analysis (Figure 1E) identified key hub genes, including TNF, IL6, IL1B, TLR4, STAT3, and PTGS2, which play critical roles in inflammatory signaling. These hub genes act as central regulators within the network and are strongly associated with PCOS pathophysiology. For example, TNF-α and IL-6 contribute to insulin resistance, while TLR4 serves as an upstream activator of NF-κB signaling. STAT3 is involved in cytokine signaling, and PTGS2 regulates prostaglandin synthesis and inflammatory responses. Dysregulation of these genes has been widely reported in PCOS and is associated with disease severity.

## 4. Interaction Analysis (2D and 3D Visualization)

**Figure 2:**
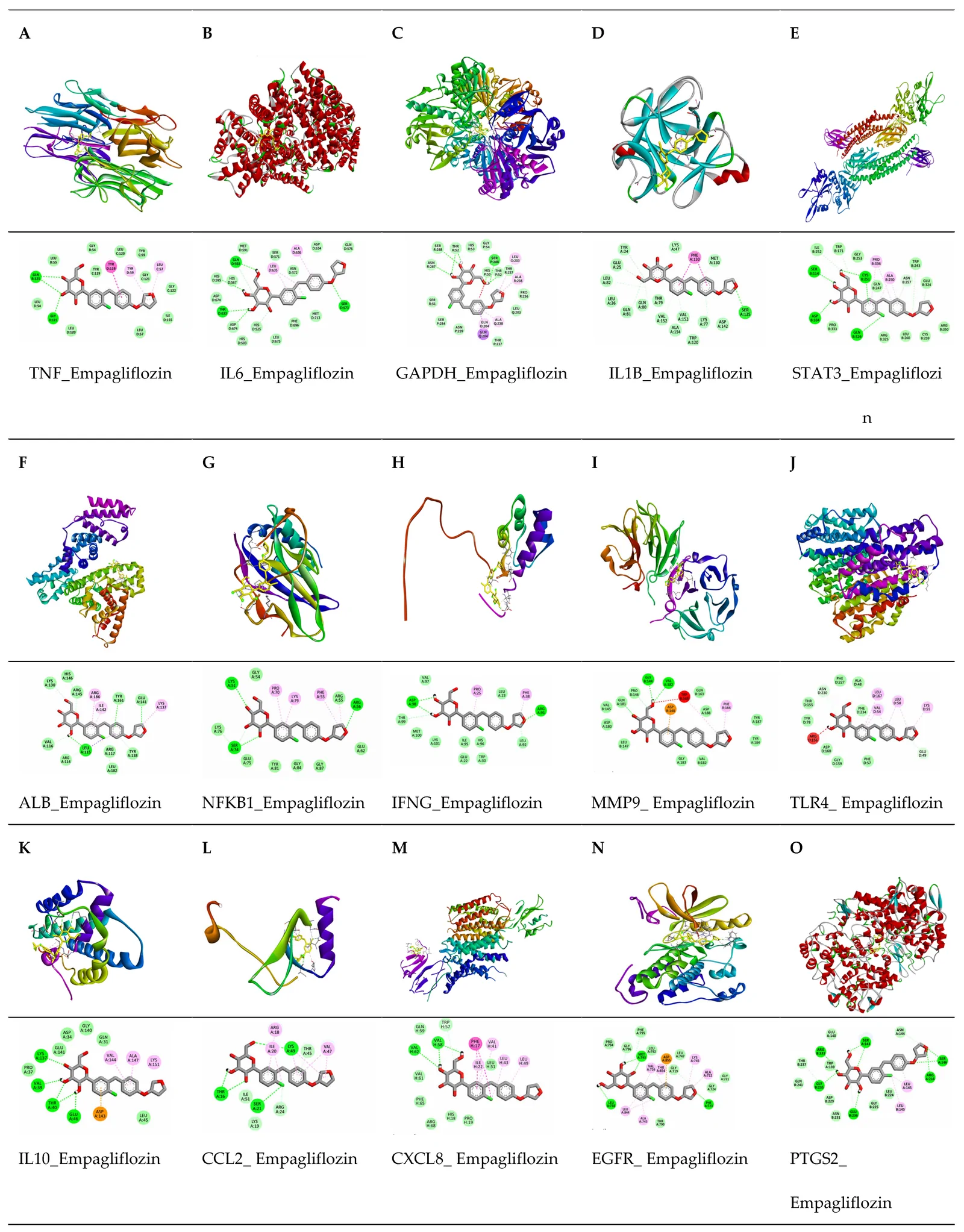
Three-dimensional (3D) binding conformations and two-dimensional (2D) interaction profiles of empagliflozin with selected target proteins. (**A**) TNF–empagliflozin; (**B**) IL6–empagliflozin; (**C**) GAPDH–empagliflozin; (**D**) IL1B–empagliflozin; (**E**) STAT3–empagliflozin; (**F**) ALB–empagliflozin; (**G**) NFKB1–empagliflozin; (**H**) IFNG–empagliflozin; (**I**) MMP9–empagliflozin; (**J**) TLR4–empagliflozin; (**K**) IL10–empagliflozin; (**L**) CCL2–empagliflozin; (**M**) CXCL8–empagliflozin; (**N**) EGFR–empagliflozin; and (**O**) PTGS2–empagliflozin. The 2D interaction profiles illustrate the interactions between empagliflozin and the respective target residues. Interaction types are color-coded as follows: van der Waals interactions are shown in light green; conventional hydrogen bonds in bright green; carbon–hydrogen bonds in pale green; unfavorable donor–donor interactions in red; π-cation interactions in orange; π-stacked interactions in magenta; π-alkyl interactions in light pink; π-donor hydrogen bonds in pale green; and π-sigma interactions in purple.

The interaction analysis (Figure 2) revealed that empagliflozin forms stable complexes with target proteins through various non-covalent interactions, including hydrogen bonds, hydrophobic interactions, van der Waals forces, and π–π stacking interactions. Hydrogen bonds contribute to binding specificity, while hydrophobic interactions enhance stability within the binding pocket. The presence of multiple interaction types suggests that empagliflozin can effectively stabilize within the active sites of target proteins. In particular, interactions observed in PTGS2 indicate potential inhibition of prostaglandin synthesis, while interactions in TLR4 suggest interference with upstream inflammatory signaling. These findings are consistent with previous studies demonstrating that SGLT2 inhibitors can suppress inflammatory pathways and reduce cytokine production.

## 5. Molecular Docking Analysis

The molecular docking results (Table 2) demonstrated that empagliflozin exhibits strong binding affinities toward multiple target proteins, with values ranging from −6.5 to −9.2 kcal/mol. Among these, PTGS2 (−9.0 kcal/mol) and TLR4 (−8.8 kcal/mol) showed particularly strong interactions, suggesting that they may serve as key therapeutic targets. Although GAPDH exhibited the lowest binding energy, its role as a housekeeping protein limits its relevance in disease-specific targeting. In contrast, PTGS2 and TLR4 are directly involved in inflammatory pathways, making them more biologically significant. The strong binding affinity indicates stable ligand–protein interactions, which are essential for effective inhibition of protein activity [27]. Compounds with binding energies below −7.0 kcal/mol are generally considered to have good binding stability and potential biological activity.

## 6. Molecular Dynamics Simulation Analysis

To further validate the molecular docking results and evaluate the dynamic stability of empagliflozin within the binding pockets of the identified hub targets, MD simulations were performed for 50 ns on the TLR4–empagliflozin and PTGS2–empagliflozin complexes [28]. The structural stability and conformational behavior of both complexes were assessed through RMSD, SASA, radius of gyration (Rg), and RMSF analyses [29].

**Figure 3:**
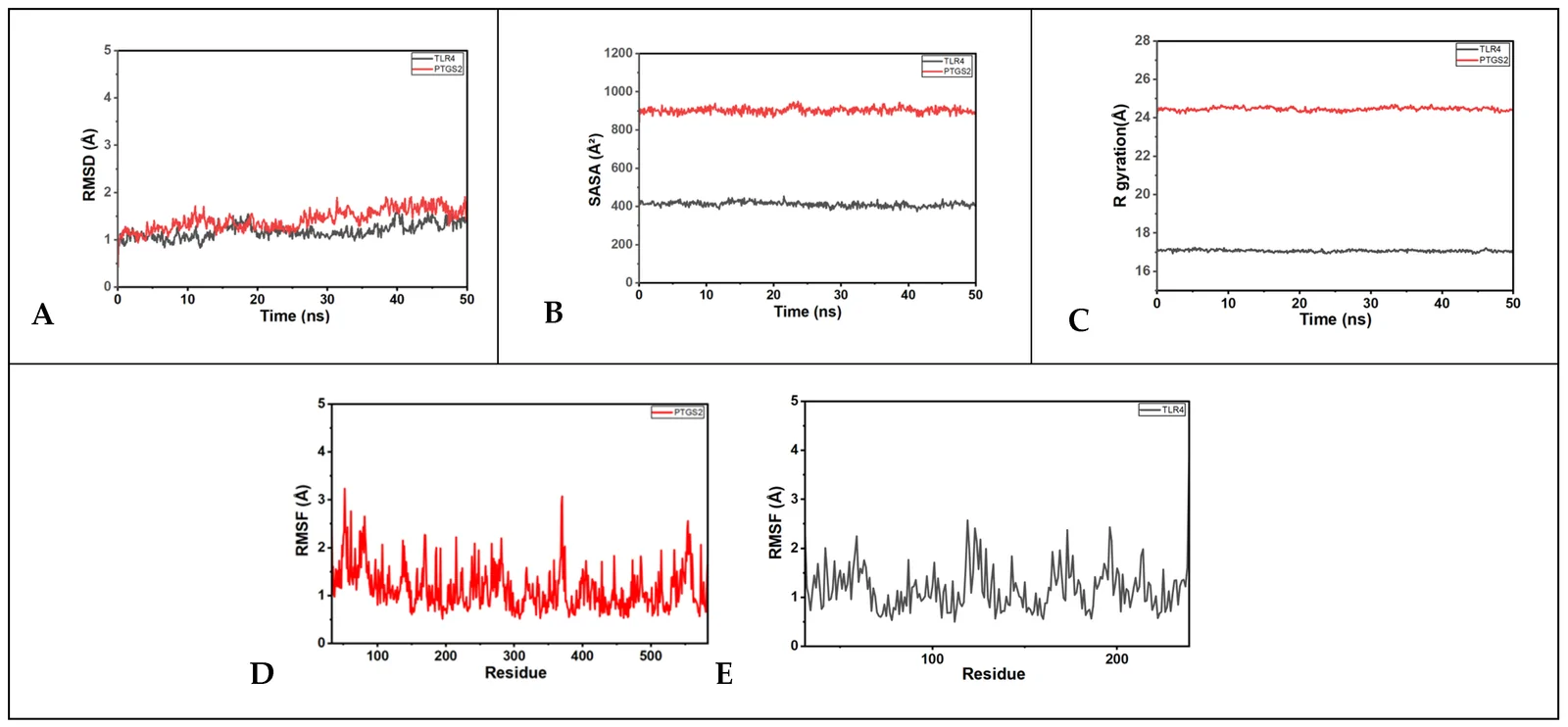
Molecular dynamics simulation analysis of empagliflozin in complex with TLR4 and PTGS2 over a 50 ns simulation period. (**A**) Root mean square deviation (RMSD) profiles showing the structural stability of the TLR4–empagliflozin and PTGS2–empagliflozin complexes. (**B**) Solvent-accessible surface area (SASA) analysis indicating changes in protein surface exposure during the simulation. (**C**) Radius of gyration (Rg) plots representing the compactness and folding stability of the protein–ligand complexes. (**D**) Root mean square fluctuation (RMSF) of PTGS2 residues illustrating residue-level flexibility throughout the simulation. (**E**) RMSF profile of TLR4 residues showing local conformational fluctuations upon empagliflozin binding.

The RMSD trajectories of both complexes are presented in Figure 3. The TLR4–empagliflozin complex exhibited a relatively stable RMSD profile throughout the simulation period, fluctuating between 0.9 and 1.5 Å. After a brief equilibration phase during the first 10 ns, the complex maintained a consistent RMSD trend with only minor deviations, indicating a stable binding conformation. Similarly, the PTGS2–empagliflozin complex showed acceptable structural stability, with RMSD values ranging between approximately 1.0 and 1.9 Å. Although PTGS2 displayed slightly higher fluctuations than TLR4 during the later stages of the simulation, the RMSD values remained below 2.0 Å throughout the 50 ns trajectory. Importantly, neither complex exceeded the commonly accepted stability threshold of 2.5 Å, suggesting that empagliflozin remained firmly accommodated within the active sites of both proteins during the simulation period.

The compactness of the protein structures was further evaluated using the radius of gyration (Rg) parameter. As shown in Figure 3, the TLR4–empagliflozin complex maintained a nearly constant Rg value of approximately 17.0 Å throughout the simulation, indicating preservation of its overall structural compactness. Likewise, the PTGS2–empagliflozin complex exhibited a stable Rg profile centered at approximately 24.4–24.6 Å, with only minimal oscillations. The absence of significant changes in Rg values suggests that no major unfolding or structural expansion occurred during the simulation, supporting the conformational stability of both protein–ligand systems.

Furthermore, SASA analysis was conducted to investigate changes in protein surface exposure during the simulation. The TLR4 complex displayed a stable SASA value of approximately 390–420 Å^2^, while the PTGS2 complex maintained a relatively constant SASA profile of approximately 880–930 Å^2^. Only minor fluctuations were observed in both systems, indicating that ligand binding did not induce substantial alterations in protein surface accessibility. The stable SASA trajectories suggest that the complexes preserved their structural integrity and did not undergo significant expansion or contraction throughout the simulation.

The flexibility of individual amino acid residues was assessed through RMSF analysis. The TLR4–empagliflozin complex demonstrated generally low residue fluctuations, with most residues exhibiting RMSF values below 2.0 Å. A few localized regions displayed moderate flexibility, particularly around residues 120–140 and 170–210, which may correspond to loop regions naturally involved in protein motion. Similarly, the PTGS2–empagliflozin complex exhibited RMSF values predominantly below 2.0 Å, with several isolated peaks reaching approximately 3.0 Å. These fluctuations were mainly restricted to terminal or loop regions and did not affect the overall stability of the protein core. The low RMSF values observed across both complexes indicate that empagliflozin binding contributes to maintaining a stable structural framework throughout the simulation.

Overall, the MD simulation results demonstrated that empagliflozin formed stable complexes with both TLR4 and PTGS2. The consistently low RMSD values, stable Rg and SASA profiles, and limited residue fluctuations collectively indicate favorable conformational stability and sustained protein–ligand interactions. These findings further support the molecular docking results and suggest that empagliflozin may exert anti-inflammatory effects in PCOS through stable modulation of TLR4-mediated inflammatory signaling and PTGS2-associated prostaglandin pathways.

## 7. Mechanistic Insight into Anti-Inflammatory Activity

By integrating the results of network pharmacology and molecular docking, a comprehensive mechanism can be proposed in which empagliflozin modulates the TLR4/NF-κB signaling pathway. Activation of TLR4 leads to downstream activation of NF-κB, which promotes the production of pro-inflammatory cytokines such as TNF-α, IL-6, and IL-1β. By inhibiting this signaling cascade, empagliflozin may reduce inflammation and improve metabolic dysfunction in PCOS. This mechanism aligns with experimental findings showing that SGLT2 inhibitors can suppress NF-κB activation and decrease inflammatory cytokine levels.

## 8. Clinical Implications and Study Limitations

From a clinical perspective, the findings of this study suggest that empagliflozin has potential as a repurposed therapeutic agent for PCOS. Unlike conventional therapies such as metformin, which primarily target insulin resistance, empagliflozin may simultaneously modulate metabolic and inflammatory pathways, offering a more comprehensive therapeutic approach. This dual action is particularly advantageous in PCOS, where multiple pathological mechanisms coexist. Recent clinical studies have reported that SGLT2 inhibitors can improve metabolic parameters and reduce body weight in patients with PCOS, further supporting their therapeutic potential [14].

However, several limitations should be acknowledged. This study is based entirely on in silico approaches, which cannot fully replicate the complexity of biological systems. Molecular docking provides a static representation of ligand–protein interactions and does not account for dynamic conformational changes. Therefore, biochemical and cellular assays, followed by appropriate animal studies and ultimately well-designed clinical studies, are required to determine whether the predicted interactions translate into functional anti-inflammatory effects and clinically meaningful therapeutic benefits.

## 9. Conclusions

This study revealed the anti-inflammatory mechanisms of empagliflozin in PCOS through an integrated network pharmacology, molecular docking, and MD approach. PTGS2 and TLR4 were identified as key targets involved in TNF and NF-κB signaling pathways. Molecular docking demonstrated favorable binding affinities of empagliflozin toward both targets, while MD simulations confirmed the stability of the TLR4–empagliflozin and PTGS2–empagliflozin complexes throughout the 50 ns simulation period. These findings suggest that empagliflozin may attenuate PCOS-associated inflammation by modulating the TLR4–NF-κB–PTGS2 signaling axis. Overall, this study provides computational evidence supporting the potential repurposing of empagliflozin as a therapeutic agent for inflammation-related manifestations of PCOS, although further experimental and clinical validation is required.

## Figures and Tables

**Table 1:** Predicted pharmacological activities and associated probabilities (Pa and Pi) of empagliflozin.

Ligand	Pa	Pi	Activity	Key Word
empagliflozin	0.850	0.004	Antidiabetic	Antidiabetic
	0.290	0.033	Antidiabetic (type 2)	
	0.203	0.121	Antidiabetic symptomatic	
	0.300	0.023	Antioxidant	Antioxidant
	0.416	0.087	Anti-inflammatory	
	0.159	0.150	Non-steroidal anti-inflammatory agent	
	0.416	0.087	Anti-inflammatory	Anti-inflammatory
	0.159	0.150	Non-steroidal anti-inflammatory agent	
	0.244	0.236	Insulin promoter	Insulin

**Table 2:** Molecular docking results of empagliflozin with selected target proteins.

Ligand	PDB ID	Binding Affinity (kcal/mol)	RMSD (Å)
TNF_Empagliflozin	2AZ5	−8.3	0.0
IL6_Empagliflozin	5SFK	−7.9	0.0
GAPDH_Empagliflozin	1U8F	−9.2	0.0
IL1B_Empagliflozin	1TWM	−7.4	0.0
STAT3_Empagliflozin	6TLC	−7.8	0.0
ALB_Empagliflozin	7VR0	−8.1	0.0
NFKB1_Empagliflozin	8TQD	−7.5	0.0
IFNG_Empagliflozin	2E80	−7.7	0.0
MMP9_Empagliflozin	1ITV	−7.2	0.0
TLR4_Empagliflozin	5UC8	−8.8	0.0
IL10_Empagliflozin	1N1F	−7.3	0.0
CCL2_Empagliflozin	1DOL	−6.5	0.0
CXCL8_Empagliflozin	8YNF	−8.3	0.0
EGFR_Empagliflozin	2ITY	−8.3	0.0
PTGS2_Empagliflozin	5F19	−9.0	0.0

## Data Availability

The original contributions presented in this study are included in the article. Further inquiries can be directed to the corresponding author.
